# Effect of dietary flaxseed oil level on the growth performance and fatty acid composition of fingerlings of rainbow trout, *Oncorhynchus mykiss*

**DOI:** 10.1186/2193-1801-2-1

**Published:** 2013-01-03

**Authors:** Ali Masiha, Nasrollah Mahboobi Soofiani, Eisa Ebrahimi, Mahdi Kadivar, Mohammad Reza Karimi

**Affiliations:** 1 Department of Natural Resources, Isfahan University of Technology, Isfahan, 84156-83111 Iran; 2 Department of Food Science, College of Agriculture, Isfahan University of Technology, Isfahan, 84156-83111 Iran

**Keywords:** Body composition, Fatty acid, Fish oil replacement, Rainbow trout, Vegetable oil

## Abstract

This study evaluated the suitability of flaxseed oil as a source of supplemental dietary lipid for fingerlings of rainbow trout (*Oncorhynchus mykiss*). Triplicate groups of the 30 fingerlings held under identical culture conditions were fed twice daily by iso-nitrogenous, iso-calorific and iso-lipidic diets for 8 weeks. Experimental diets consisted of 30.2% protein, 18.6 kJ g^-1^ energy and 16.5% lipid from fish oil (FO), flaxseed oil (FxO) and 1:1 blends of the oils (FFxO). Moisture, ash, protein, final body weight, specific growth rate, weight gain, feed conversion ratio, survival and hepatosomatic index were not affected by treatments but the percent of lipids was significantly highest in fish fed the flaxseed oil diet (FxOD). The condition factors of fingerlings reared on FxOD and fish and flaxseed oils diet (FFxOD) were significantly lower than those fed the fish oil diet (FOD). Protein efficiency ratio (PER) was significantly higher than those fed the FOD and FFxOD. Whole body fatty acid compositions mirrored those of diet treatments. The highest amounts of highly unsaturated fatty acids (HUFAs) were detected in fish fed 100% FO, which was significantly different from other treatments. In all treatments polyunsaturated fatty acids/saturated fatty acids (PUFAs/SFAs) and n-6/n-3 ratios were higher than 0.45 and lower than 4, respectively. Present results indicate the fingerlings can be reared on diets in which FO has been replaced with FxO, with no significant effects on fish performance.

## Introduction

In the course of just a few decades, fish farming has developed into a highly productive and efficient industry to produce animal protein for human consumption. In addition to good growing conditions, a prerequisite for productivity and economic sustainability in fish farming can be a reliable supply of effective feeds. For various reasons, fish meal and fish oil have historically been the dominant raw materials in the production of fish feeds. Due to the development of more energy dense feed types as well as general growth of the aquaculture industry, a significant proportion of the total global fish oil is used for its feed preparation. A lipid requirement equal to 100% of the world’s total fish oil production is estimated by the year 2010 (New, 1999).

While marine oils are superior in their fatty acid composition they also contain a variety of toxic compounds including polychlorinated dibenzo-p-dioxins (PCDD), polychlorinated dibenzofurans (PCDF) and dioxin-like polychlorinated biphenyls (DL-PCB), particularly the non-ortho and mono-ortho substituted PCBs (Jacobs *et al*., [Bibr CR25_64][Bibr CR26_64]; Hites *et al*., [Bibr CR21_64][Bibr CR22_64]). These compounds are suspected to be carcinogenic and immunosuppressive in humans (Birnbaum and Tuomisto, 2000; Baccarelli *et al*., 2002; Van Den Heuvel *et al*., 2002).

It is also well-known that lipid oxidation is one of the major concerns in fish-derived food products. Polyunsaturated fatty acids (PUFAs) are more easily oxidized than saturated fatty acids (SFAs), and therefore, food products enhanced with the PUFAs n-3 are also more prone to lipid oxidation. There is potential human health risks associated with increased consumption of oxidized PUFAs n-3 products (Fritsche and Johnston, 1990; Kubow, 1993). Another important factor to limit a more common use of PUFAs n-3 enhanced food products is the development of off-flavors following lipid oxidation that may be offensive to consumers (Waagbø *et al*., 1993).

While it is obvious that a substitute must be found, replacing fish oil in diets has its own difficulties as most of the vegetable oils are relatively poor sources of n-3 fatty acids. Exceptions to this are flaxseed and canola oils which are rich in alpha linolenic acid (18:3n-3) (53% and 12%, respectively) (NRC, 1993). However, these oils are devoid of longer chain n-3 highly unsaturated fatty acids (HUFAs n-3) and their inclusion in trout diets results in a significant decrease in the tissue levels of eicosapentaenoic acid (20:5n-3, EPA) and docosahexaenoic acid (22:6n-3, DHA) (Bell *et al*., [Bibr CR4_64][Bibr CR5_64]). Moreover, enhancement of omega-3 fatty acid content in rainbow trout fillet was observed in farmed rainbow trout and brook trout as results of flaxseed oil inclusion in diet (Chen *et al*., 2006; Simmons *et al*., 2011).

Freshwater fish are capable of converting C18 PUFAs to the longer chain C20 and C22 PUFAs (Henderson and Tocher, 1987) which are the functionally essential fatty acids in vertebrates (Lauritzen *et al*., 2001).

Several studies conducted on freshwater fish indicated that vegetable oils can successfully replace fish oil in the feed without affecting their survival and growth (Wonnacott *et al*., 2004; Subhadra *et al*., 2006). Caballero *et al*., (2002) reported that in rainbow trout (*Oncorhynchus mykiss*) up to 80–90% of vegetable oils e.g. soybean, rapeseed, olive, and palm oils can be used without compromising fish growth. It has also been reported that partial replacement of fish oil by vegetable oils such as rapeseed, soybean, flaxseed or palm oils in fish feeds had no negative impacts on growth and survival of Atlantic salmon (*Salmo salar*) (Rosenlund *et al*., 2001), brook char (*Salvelinus fontinalis*) (Guillou *et al*., 1995), gilthead sea bream (*Sparus aurata*), European sea bass (*Dicentrarchus labrax*) (Izquierdo *et al*., 2003) and rainbow trout (Greene and Selivonchick, 1990; Caballero *et al*., 2002).

The aim of the present study was to evaluate the effects of fish oil replacement with flaxseed oil (relatively easily obtained and low priced oil) on growth, feed conversion ratio and fillet fatty acid composition of fingerlings of rainbow trout.

## Materials and methods

270 rainbow trout fingerlings with a mean initial body weight of 16.5 ± 0.5 g were purchased from Cheshmeh Dimeh fish hatchery (Shahre kord, Chaharmahal and Bakhtiari, Iran) and used in this study. Prior to the start of the experiment the fish were acclimatized to the new environmental conditions and the commercial diet (SFT2 of Chineh feed production factory, Tehran, Iran) for a two week period within a semi re-circulating system.

### Experimental diets

Three iso-nitrogenous, iso-calorific and iso-lipidic purified experimental diets were formulated from 100% fish oil (FO), 100% flaxseed oil (FxO) and 1:1 blends of the two oils (FFxO). The nutritional content and Fatty acid composition of the oils and experimental diets are presented in Tables 1 and 2, respectively. Diets were prepared and stored according to Abery *et al*., (2002) and De Silva *et al*., (2002).

**Table 1:** **Ingredient (%)**, **proximate composition (% wet weight) and energy (kJ g**^**−1**^**) of the experimental diets**

	FOD	FxOD	FFxOD
Fish Meal	58	58	58
Soybean Meal	20	20	20
Wheat Meal	8.6	8.6	8.6
Fish Oil	8	0	4
Flaxseed Oil	0	8	4
Vitamin premix*	2	2	2
Mineral premix**	1.5	1.5	1.5
Lysine	0.07	0.07	0.07
Methionine	0.13	0.13	0.13
Choline chloride	0.2	0.2	0.2
Molasses	1	1	1
Salt	0.5	0.5	0.5
*Proximate composition*			
Moisture	9.80	9.10	9.63
Ash	14.59	14.76	15.29
Crude protein	30.66	30.15	29.85
Crude lipid	16.09	16.52	16.81
Crude fiber	2.16	2.42	2.01
NFE***	26.70	27.05	26.41
Energy****	18.56	18.71	18.57

Diet abbreviations, FOD: 100% fish oil; FxOD: 100% Flaxseed oil; FFxOD: 50% fish oil and 50% flaxseed oil.

*Contains (mg kg^-1^ food): E (30), K (3), niacin (40), thiamine (2), riboflavin (7), pyridoxine (3), folacin (1.5), pantothenic acid (18), biotin (0.7) and cyanocobalamin (0.18).

**Contains (mg kg^-1^ food): Mg (100), Zn (60), Fe (40), Cu (5), Co (0.1), I (1) and Antioxidant (100).

***NFE: nitrogen free extract, calculated by difference (100 – moisture – ash – crude protein – crude lipid –crude fibers).

****Calculated on the basis of 23.6, 39.5 and 17.2 kJ g^−1^ of protein, fat and carbohydrate, respectively.

**Table 2:** **Fatty acid composition (percentage of total fatty acids) of the oils and experimental diets**

Fatty acid	Fish Oil	Flaxseed Oil	FOD	FxOD	FFxOD
14:0	0.06	-	0.07	0.04	0.05
15:0	0.32	-	0.23	0.09	0.17
16:0	20.73	6.79	22.71	16.48	19.80
17:0	0.72	0.11	0.72	0.51	0.67
18:0	4.16	4.48	5.85	6.04	6.20
19:0	2.89	1.23	2.94	2.31	2.53
21:0	0.18	-	0.25	0.24	0.29
22:0	0.24	0.04	0.20	0.13	0.15
23:0	0.21	0.57	0.22	0.30	0.33
24:0	0.18	-	0.14	0.08	0.11
SFAs	29.68	13.22	33.32	26.22	30.29
14:1	3.77	0.26	3.11	1.37	2.26
15:1	0.80	0.07	0.71	0.38	0.56
16:1n-7	5.24	0.34	4.92	2.59	3.86
17:1	0.71	0.06	0.65	0.27	0.41
18:1n-9	33.57	34.19	38.79	41.75	40.50
24:1n-9	0.44	-	0.41	0.14	0.26
MUFAs	44.52	34.92	48.59	46.50	47.85
18:2n-6	0.37	0.38	0.42	0.39	0.61
18:3n-6	0.05	-	0.04	0.05	0.05
20:2n-6	2.48	0.13	1.21	0.24	0.68
20:3n-6	0.18	-	0.20	0.25	0.21
20:4n-6	0.02	-	0.06	0.03	0.05
22:2n-6	0.72	-	0.65	0.37	0.52
22:5n-6	0.36	-	0.30	0.18	0.21
PUFAs n-6	4.18	0.50	2.88	1.51	2.32
18:3n-3	2.07	51.36	4.58	21.93	12.64
18:4n-3	0.32	-	0.60	0.71	0.84
20:3n-3	0.05	-	0.61	0.05	0.05
20:5n-3	5.90	-	2.95	0.73	1.69
22:5n-3	0.48	-	0.36	0.22	0.28
22:6n-3	12.82	-	6.65	2.16	4.05
PUFAs n-3	21.62	51.36	15.75	25.80	19.55
HUFAs n-3	18.72	-	9.60	2.89	5.74

- not detected.

See Table 1 for diet abbreviations.

### Husbandry

This study was conducted indoors in a thermostatically controlled room. Fish were housed in nine 100 L fiberglass circular rearing tanks in a semi re-circulating system with an in-line oxygen generator and a physical and biological treatment plant (flow rate of 6 L min^−1^). During experiment, fish were kept under a 12-h light: 12-h dark cycle. The experiment was conducted at 13.6 ± 1.3°C, water quality parameters were measured every second day using Aquamerck test kits (Merck, Darmstadt, Germany) with a mean pH of 7.3 ± 0.2 and levels of ammonia and nitrate below 0.1 mg L^−1^.

### Experimental protocol

Two hundred and seventy individually weighed and measured rainbow trout (*O*. *mykiss*) fingerlings were randomly distributed into nine 100 L fiberglass tanks (30 fish per tank) and randomly assigned to one of the 3 different experimental diets (3 replicates for each experimental diet). Fish were fed twice daily at approximately 08.30 and 17.00 h to apparent satiation for a period of 56 days. At the end of the experiment a sample of 18 fish (2 fish per replicate) was taken and anesthetized in excess anesthetic (Benzocaine 0.5 mg L^−1^) for both body composition and fatty acid profile analysis.

### Chemical analysis

Fishes allocated for flesh analysis were filleted (denuded of skin and bone) and stored at −20°C until used for fillet proximate analysis. Fishes allocated for fatty acids analysis were stored at −80°C. Proximate analysis was conducted using standard procedures (AOAC, 1990), percentage moisture (dried at 80°C to constant weight), protein (Kjeldahl nitrogen; N × 6.25) in an automated Kjeltech (Model 2300, Tecator, Sweden), total lipid by chloroform/methanol extraction (2:1 v/v) (Folch et al., 1957) as modified by Ways and Hanahan (1964) and ash by incineration in a muffle furnace (Model WIT, C & LTetlow, Australia) at 550°C for 18 h. Fatty acid analysis was carried out on each of the added dietary oils, experimental diets and fillet samples from each of the replicates. Fatty acid methyl esters (FAMEs) were prepared from aliquots of total lipids by acid catalyzed transmethylation with sulfuric acid in methanol overnight at 50°C (Christie, 1982). FAMEs were purified by TLC using hexane/diethyl ether/acetic acid (85:15:15 v/v/v) as solvent (Tocher and Harvie, 1988). Separation of FAMEs was carried out in a Gas Chromatograph system (Agilent Technologies, 6890 N, USA) equipped with a flame ionization detector (FID), and a cross-linked silica capillary column HP-88 (100 m, 250 μm ID, 0.2 μm film thickness), on-column injection and using helium as the carrier gas with a flow rate of 1.1 ml min^-1^. The column was programmed for an initial temperature of 140°C held for 5 min, rising at a rate of 4°C min^-1^ to the final temperature of 240°C and held for 10 min. Injector and detector temperatures were 230°C and 260°C, respectively. The flow rates of compressed air and hydrogen were 300 ml min^-1^ and 30 ml min^-1^, respectively. Identification and quantification of FAMEs were based on the comparison of the sample retention time with known standards (Sigma Chemicals, St. Louis, USA).

### Statistical analysis

Mean values and standard deviation for each parameter measured for all treatments were calculated first. The results were subjected to a one-way ANOVA to test the effect of the replacement of vegetable oil blends on fish performance. Data were analyzed using statistical packages SPSS v15 (SPSS Inc., Chicago, IL, USA). Differences between means were compared using Duncan’s multiple range test at significance of differences (*P* < 0.05) among dietary treatments. Linear regression analyses were performed between dietary and fillet fatty acid concentrations.

## Results

### Growth

The mean final body weight (MFBW) of fingerlings of rainbow trout (*O*. *mykiss*) ranged from 56.6 ± 8.0 to 58.5 ± 14.6 for FxOD and fish and flaxseed oils diet (FFxOD) treatments, respectively. The differences between the MFBW of fish receiving different diets were not significant. Similarly, no significant differences were observed between survival rate, specific growth rate, weight gain, feed conversion ratio and hepatosomatic index. The condition factor of fish reared on FxOD and FFxOD were significantly (*P* < 0.05) lower than those fish fed the FOD. The protein efficiency ratio (PER) was highest in fish fed the FxOD and significantly (*P* < 0.05) higher than those fed with FOD and FFxOD (Table 3).

**Table 3:** **Mean (±SD) of growth, feed utilisation and other body parameters of rainbow trout reared on the experimental diets**

	FOD	FxOD	FFxOD
MIBW	16.12 ± 0.27	16.30 ± 0.78	16.74 ± 0.33
MFBW	58.05 ± 6.98	56.56 ± 8.05	58.52 ± 14.58
CF	1.22 ± 0.10^a^	1.15 ± 0.11^b^	1.12 ± 0.10^b^
SGR	2.29 ± 0.02	2.22 ± 0.06	2.23 ± 0.08
WG	260.09 ± 4.25	246.71 ± 10.78	249.54 ± 15.23
FCR	0.90 ± 0.17	1.01 ± 0.05	0.99 ± 0.30
SR	100.00 ± 0.00	100.00 ± 0.00	100.00 ± 0.00
HIS	1.21 ± 0.13	1.14 ± 0.06	1.15 ± 0.06
PER	1.92 ± 0.22^b^	3.12 ± 0.31^a^	2.37 ± 0.16^b^

Values in the same row with the same superscripts are not significantly different (*P* > 0.05).

See Table 1 for diet abbreviations.

MIBW (g): Mean initial body weight, MFBW (g): Mean final body weight

CF: Condition factor =100 × (final weight (g)) × (fork length (cm))^−3^.

SGR (%day^-1^): Specific growth rate = [Ln(final weight) − Ln(initial weight)] × (number of days)^−1^ × 100.

WG (%): Weight Gain = (final weight − initial weight) × (initial weight^)−1^ × 100

FCR: Feed conversion ratio = (dry feed fed) × (wet weight gain)^−1^.

SR (%): Survival rate = number of fish in each group remaining on day 56 − (initial number of fish)^-1^ × 100.

HSI (%): Hepatosomatic index = (weight of liver) × (total fish weight)^−1^ × 100.

PER: Protein efficiency ratio = (final weight − initial weight) × (mass of protein fed)^−1^.

### Fillet proximate composition

Results of the proximate analysis of fillet of fish receiving the different dietary treatments are tabulated in Table 4. No significant differences between percent moisture, ash and protein content of fish fed the experimental diets were observed, but the percent of lipid content was highest in fish fed the FxOD which was significantly (*P* < 0.05) higher than lipid content of fish fed on FFxOD.

**Table 4:** **Fillet proximate compositions (mean ± SD) of rainbow trout reared on different diets**, **(% wet weight)**

	Initial*	FOD	FxOD	FFxOD
Moisture	78.44 ± 0.61	76.96 ± 0.20	76.48 ± 1.02	77.39 ± 0.41
Ash	1.34 ± 0.03	1.28 ± 0.05	1.36 ± 0.06	1.28 ± 0.08
Protein	15.29 ± 0.81	17.77 ± 0.16	18.55 ± 1.15	16.49 ± 1.30
Lipid	2.93 ± 0.20	3.46 ± 0.27^a^	3.48 ± 0.14^a^	2.90 ± 0.28^b^

See Table 1 for diet abbreviations.

*Statistics not performed on the initial sample.

Values in the same row with the same superscripts are not significantly different (*P* > 0.05).

### Fillet fatty acid composition

The major fatty acid classes (SFAs, MUFAs and PUFAs) found in the highest concentration were palmitic, oleic, α-linolenic acids along with DHA, respectively (Table 5). The level of SFAs was observed in higher (*P* < 0.05) concentrations for fish fed the FOD compared to fish fed the FxOD and FFxOD. Levels of MUFAs ranged from 47.4 ± 0.5 (FxOD) to 53.0 ± 0.4 (FOD) and were observed to be significantly higher in fish fed the FOD. The fillet of fish fed the FOD and FxOD were particularly rich in oleic acid (44.8 ± 0.4%) and α-linolenic acid (19.3 ± 0.4%), respectively. DHA and arachidonic acid levels were found in higher concentrations in the fillet than in the diets. The highest level of EPA and DHA was observed in fish fed the FOD (*P* < 0.05). However, DHA was found in high concentrations within all of the dietary treatments, ranging from 5.7 ± 0.4% (FxOD) to 10.7 ± 0.4% (FOD). The level of n-3 fatty acids was higher in the fillet than the diet for each of the treatments, but the level of n-6 fatty acids was higher in the fillet than the diet only for FxOD and FFxOD, with n-6/n-3 ratios ranging from 0.12 ± 0.00 to 0.16 ± 0.02 in the fillet. The highest HUFAs n-3 concentrations (*P* < 0.05) were found in fish fed the FOD (12.8 ± 0.4%), while the lowest value was observed in fish fed the FxOD (6.6 ± 0.4%).

**Table 5:** **Fillet fatty acid composition (percentage of total fatty acids) of rainbow trout reared on the different diets (mean ± SD)**

	Initial	FOD	FxOD	FFxOD
14:0	0.03	0.04 ± 0.01	0.03 ± 0.00	0.03 ± 0.01
15:0	0.09	0.16 ± 0.01^a^	0.07 ± 0.01^c^	0.10 ± 0.01^b^
16:0	10.11	17.45 ± 0.29^a^	12.46 ± 0.12^c^	14.42 ± 0.26^b^
17:0	0.14	0.50 ± 0.01^a^	0.15 ± 0.02^c^	0.37 ± 0.00^b^
18:0	2.90	4.46 ± 0.10^a^	4.59 ± 0.07^a^	4.27 ± 0.06^b^
19:0	3.07	3.72 ± 0.45^a^	2.10 ± 0.01^c^	2.94 ± 0.10^b^
21:0	0.21	0.19 ± 0.06	0.26 ± 0.02	0.19 ± 0.04
22:0	0.77	0.55 ± 0.06	0.56 ± 0.08	0.56 ± 0.04
23:0	0.72	0.28 ± 0.02^c^	0.90 ± 0.08^a^	0.67 ± 0.02^b^
24:0	0.41	0.41 ± 0.03^b^	0.72 ± 0.15^a^	0.60 ± 0.03^a^
SFAs	18.44	27.74 ± 0.53^a^	21.84 ± 0.20^c^	24.15 ± 0.25^b^
14:1	1.42	2.25 ± 0.08^a^	1.10 ± 0.03^c^	1.56 ± 0.05^b^
15:1	0.17	0.52 ± 0.02^a^	0.27 ± 0.00^c^	0.36 ± 0.01^b^
16:1n-7	1.77	4.49 ± 0.09^a^	2.35 ± 0.03^c^	3.28 ± 0.09^b^
17:1	0.18	0.48 ± 0.03^a^	0.17 ± 0.13^b^	0.36 ± 0.02^a^
18:1n-9	61.25	44.85 ± 0.45^a^	43.25 ± 0.63^b^	44.71 ± 0.15^a^
24:1n-9	0.10	0.40 ± 0.03^a^	0.27 ± 0.05^b^	0.32 ± 0.01^b^
MUFAs	64.88	52.99 ± 0.38^a^	47.40 ± 0.52^c^	50.60 ± 0.02^b^
18:2n-6	0.71	0.41 ± 0.05	0.52 ± 0.09	0.42 ± 0.02
18:3n-6	0.05	0.06 ± 0.00	0.06 ± 0.01	0.06 ± 0.02
20:2n-6	0.88	0.89 ± 0.06^c^	1.54 ± 0.12^a^	1.11 ± 0.04^b^
20:3n-6	0.13	0.09 ± 0.01	0.07 ± 0.03	0.08 ± 0.02
20:4n-6	0.86	0.13 ± 0.03	0.14 ± 0.01	0.13 ± 0.01
22:2n-6	0.43	0.77 ± 0.07^a^	0.65 ± 0.03^b^	0.74 ± 0.02^a^
22:5n-6	0.33	0.24 ± 0.03	0.20 ± 0.03	0.22 ± 0.01
PUFAs n-6	3.40	2.60 ± 0.24^b^	3.18 ± 0.23^a^	2.75 ± 0.02^b^
18:3n-3	4.27	1.94 ± 0.07^c^	19.35 ± 0.40^a^	10.60 ± 0.07^b^
18:4n-3	2.61	1.04 ± 0.04	1.01 ± 0.03	1.09 ± 0.06
20:3n-3	0.53	0.30 ± 0.03	0.31 ± 0.04	0.30 ± 0.01
20:5n-3	0.87	2.08 ± 0.10^a^	0.92 ± 0.05^c^	1.47 ± 0.01^b^
22:5n-3	0.25	0.59 ± 0.05^a^	0.26 ± 0.04^c^	0.46 ± 0.04^b^
22:6n-3	4.76	10.73 ± 0.40^a^	5.72 ± 0.39^c^	8.60 ± 0.18^b^
PUFAs n-3	13.28	16.67 ± 0.52^c^	27.57 ± 0.72^a^	22.51 ± 0.22^b^
HUFAs n-3	5.63	12.80 ± 0.45^a^	6.64 ± 0.42^c^	10.07 ± 0.19^b^
PUFAs	16.68	19.27 ± 0.66^c^	30.75 ± 0.57^a^	25.26 ± 0.24^b^
PUFAs/SFAs	0.90	0.69 ± 0.04^c^	1.41 ± 0.02^a^	1.04 ± 0.02^b^
n-6/n-3	0.26	0.16 ± 0.02^a^	0.12 ± 0.01^b^	0.12 ± 0.00^b^

See Table 1 for diet abbreviations.

Values in the same row with the same superscripts are not significantly different (*P* > 0.05).

Regression analysis was used to identify dose response relationship between dietary and fillet fatty acids. As reported in Table 6, most of the fatty acid concentrations in the fillet were linearly correlated to the dietary fatty acid concentrations.

**Table 6:** **Correlation between dietary fatty acid concentrations and fatty acid concentrations in fillet of rainbow trout fed the experimental diets for 8 weeks**

Fatty acids	Correlation coefficient (***r***)	Slope
16:0	0.974	0.719
18:0	0.451	0.201
SFAs	0.957	0.757
18:1n-9	0.861	−0.513
MUFAs	0.997	2.637
18:2n-6	0.537	−0.274
20:4n-6	0.945	−0.357
PUFAs n-6	0.986	−0.431
18:3n-3	0.999	1.001
20:5n-3	0.999	0.521
22:6n-3	0.984	1.098
PUFAs n-3	0.978	1.035
PUFAs	0.982	1.287

## Discussion

The results of the present study suggest that flaxseed oil can be used to replace fish oil without adverse effects on growth performance of rainbow trout fingerlings, as reported in other studies (Montero *et al*., 2005; Bell *et al*., 2004; Izquierdo *et al*., 2005). This was evidenced by the weight gain and feed conversion ratio which ranged from 246.7 ± 10.8% to 260.1 ± 4.2% and 0.90 ± 0.17 to 1.01 ± 0.05, respectively, with no significant differences from fish fed all experimental diets.

In agreement with previous studies (Caballero *et al*., 2002; Martino *et al*., 2002; Glencross *et al*., 2003; Turchini *et al*., 2003b), considerable differences were evident in the fatty acid composition of fish fed different lipid sources. For example, there was a high increase in the levels of α-linolenic acid in fish fed either with FxOD and/or FFxOD. As reported by other researchers, (Turchini *et al*., [Bibr CR42_64][Bibr CR43_64]; Guillou *et al*., 1995; Bell *et al*., [Bibr CR5_64][Bibr CR6_64]; Torstensen *et al*., 2004; Chen *et al*., 2006; Chen *et al*., 2008; Simmons *et al*., 2011), a high correlations are also exist between the individual fatty acids as well as MUFAs and PUFAs of a diet and the fish fillet (Table 6). There was, however, a high correlation between the amount of SFAs in the diet and SFAs in the fillet, which was not in accordance with the findings of Turchini *et al*., ([Bibr CR42_64][Bibr CR43_64]) who postulated that SFAs were not used efficiently by Murray cod (*Maccullochella peelii peelii*) as an energy source and were subsequently deposited at an optimal level in preference to the other major fatty acid classes.

It is well known that freshwater fish have a dietary requirement for n-3 and n-6 fatty acids, predominantly in the form of α-linolenic and linoleic acids (Kanazawa *et al*., 1979,1980; Guillou *et al*., 1995; Martino *et al*., 2002; Izquierdo *et al*., 2003; Tocher, 2003). In comparison to marine fish species, freshwater fish are also generally better adapted to desaturate and elongate these base fatty acids to higher homologs (Guillou *et al*., 1995; Tocher, 2003). This study observed α-linolenic acid in lower concentrations in the muscle than in the diets. It is therefore suspected that a high degree of metabolism of this fatty acid for β-oxidation and/or desaturation and elongation is taking place in fingerlings of rainbow trout (*O*. *mykiss*). This is further bolstered by the presence of n-3 desaturation and elongation enzyme products in the form of 18:4n-3 and 20:3n-3 in fish fed FxOD and FFxOD. These fatty acids were found in much lower concentrations in the diets. Likewise, fish fed the FxOD and FFxOD contained n-6 desaturation and elongation intermediates (18:3n-6 and 20:3n-6) and indicate an elongation and desaturation of linoleic acid via Δ6 desaturase. However, further desaturation of 20:3n-6 to 20:4n-6 and 20:3n-3 to EPA and ultimately DHA was shrouded by high concentrations of these fatty acids within the fillet of initial fish samples. The Department of Health of England (Committee on Medical Aspects of Food Policy 1994) recommends a minimum PUFAs/SFAs ratio of 0.45, and a maximum n-6/n-3 of 4.0. Table 5 shows that our fish in all treatments met the PUFAs/SFAs and n-6/n-3 ratios. Despite the decrease in EPA and DHA in fillet from fish fed FFxOD, the trout fillets contained a relatively rich source of these fatty acids (584 mg of EPA plus DHA) with a 200 g serving portion of the fillets from fish fed FFxOD. This meets the intake of 500 mg day^−1^ of EPA plus DHA recommended by the International Society for the Study of Fatty Acids and Lipids (Simopoulos *et al*., 1999).

## Conclusion

Present study showed the substitution of fish oil with flaxseed oil in the rainbow trout (*O*. *mykiss*) diet have been possible without any negative effects on the growth and feed conversion ratio. However, the reflection of the dietary oil source on the fillet fatty acid composition of the fish could be a potential drawback for vegetable oil substitution from a human nutritional point of view, given the decreases in levels of EPA and DHA in fish fed the vegetable oil diets. Further investigation into the benefits of other vegetable oils or indeed a blend of various vegetable oils is required in order to reduce usage of traditionally used fish oils, while simultaneously avoiding a reduction in the human health protective properties found within fish flesh.
